# Isolated third ventricle glioblastoma

**DOI:** 10.1186/s40064-016-1746-z

**Published:** 2016-02-03

**Authors:** Baran Yılmaz, Murat Şakir Ekşi, Mustafa Kemal Demir, Akın Akakın, Zafer Orkun Toktaş, Özlem Yapıcıer, Türker Kılıç

**Affiliations:** Department of Neurosurgery, Medical Faculty, Bahçeşehir University, Istanbul, Turkey; Department of Orthopedic Surgery-Spine Center, University of California at San Francisco, 500 Parnassus Avenue MU320 West, San Francisco, CA 94143-0728 USA; Department of Radiology, Medical Faculty, Bahçeşehir University, Istanbul, Turkey; Department of Pathology, Medical Faculty, Bahçeşehir University, Istanbul, Turkey

**Keywords:** Biopsy, Glioblastoma, Third ventricle, Hydrocephalus, Ventriculo-peritoneal shunt

## Abstract

**Introduction:**

Glioblastoma is the most common and the most malignant type of gliomas. Cerebral hemispheres are usual locations for gliomas. Isolated third ventricular presentation is very rare for glioblastomas. A new case of isolated third ventricular glioblastoma has been presented in this report.

**Case description:**

A 36-year-old woman was admitted to outpatient clinic with headache, blurred vision and confusion. A head CT scan and MRI had showed third ventricular mass lesion with obstructive hydrocephalus. Previous to her admission to our clinic, a ventriculo-peritoneal shunt had been inserted and her hydrocephalus had been relieved to some extent in acute settings. In our clinic, stereotactic biopsy was performed and a second ventriculoperitoneal shunt was inserted from the opposite site. Histopathological diagnosis was glioblastoma. Radiotherapy and chemotherapy were started immediately after the surgery. Patient’s hydrocephalus has resolved and she was well at post-operative 6th month.

**Discussion and evaluation:**

In differential diagnosis list of the tumors presenting in the third ventricle, there are plenty of tumors such as colloid cyst, meningioma, germinoma, craniopharyngioma, lymphoma, choroid plexus papilloma, subependymal giant cell astrocytoma, chiasmatic and hypothalamic benign astrocytoma. Ring enhancement of this region pathology is a peculiar sign for glioblastoma, yet not pathognomonic. Tumor histology is crucial to yield the final diagnosis.

**Conclusion:**

Management of obstructive hydrocephalus, making histopathological diagnosis, starting adjuvant radiotherapy and chemotherapy in isolated third ventricular glioblastomas is a safe and effective approach when we consider malignant nature and intractable progress of glioblastomas.

## Introduction

Gliomas are primary tumors of central nervous system (CNS). They are pathologically derived from astrocytes (82 %), oligodendrocytes, or both cell types (Cohen and Colman 2015). Histologically, gliomas are graded in 4 subtypes. Glioblastoma (grade 4 glial tumor) is the most common neoplasm of astrocytomas (50–60 %) (Prieto et al. 2006). Glial tumors mostly present in cerebral hemispheres (86 %), only a very small percent of them (2.2 %) present in the ventricles (Larjavaara et al. 2007). Isolated third ventricular presentation is very rare especially for glioblastoma (Table 1) (Prieto et al. 2006; Lee and Manzano 1997; Hariri et al. 2014; Vougiouklakis et al. 2006; Lejeune et al. 2000; Villani and Tomei 2000; Yasargil 1996; AlbertLasierra 1999).

**Table 1:** Isolated third ventricular glioblastoma cases described in literature

Author(s)	Number of cases	Age (years)/sex	Clinical features	Neuroradiological features	Treatment
Yasargil (1996)	4	N/A	N/A	Anterior portion of the third ventricle	Frontal interhemispheric approach
Lee and Manzano (1997)	1	59/M	Depression, anxiety disorder, urinary incontinence, disorientation, memory deficits, gait disorder	Third-ventricle ring enhanced lesion with obstructive hydrocephalus	Ventriculostomy, transcallosal partial removal of the tumor, postoperative radio- and chemo-therapy
Albert Lasierra (1999)	2	N/A	N/A	N/A	N/A
Lejeune et al. (2000)	2	N/A	N/A	N/A	N/A
Villani and Tomei (2000)	1	N/A	N/A	N/A	N/A
Vougiouklakis et al. (2006)	1	34/M	Unconsciousness	Hydocephalus, hemorrhagic third ventricle lesion	Died
Prieto et al. (2006)	1	29/F	Polydipsia, polyuria, depression	Heterogenous anterior third ventricle lesion with ring-enhancement, obstructive hydrocephalus	V-P shunt for hydrocephalus, frontal-transcortical-transventricular, subtotal removal of the lesion
Hariri et al. (2014)	1	62/F	Headache, somnolence, cognitive decline	Homogenous, non-enhancing mass with mild hydrocephalus	N/A
Present case	1	36/F	Headache, blurred vision, confusion	Heterogeneously enhanced multi-cystic lesion with obstructive hydrocephalus	V-P shunt for hydrocephalus, stereotactic biopsy from the tumor, post-operative radiotherapy and chemotherapy

*F* female, *M* male, *N/A* not available, *V*-*P* ventriculo-peritoneal

We present the first well-defined isolated third ventricular glioblastoma, which has been managed with stereotactic biopsy and adjuvant chemoradiotherapy. We discuss clinical, radiological, therapeutic aspects of this rare pathology, with a literature review.

## Case report

A 36-year-old woman was admitted to outpatient clinic with headache, blurred vision and confusion. A head CT scan and MRI showed third ventricular mass lesion with obstructive hydrocephalus. Previous to her admission to our clinic, a ventriculo-peritoneal (V-P) shunt had been inserted and her hydrocephalus had been relieved to some extent in acute settings. Thereafter, she had been referred to our clinic for management of the third ventricular lesion.

**Fig. 1:**
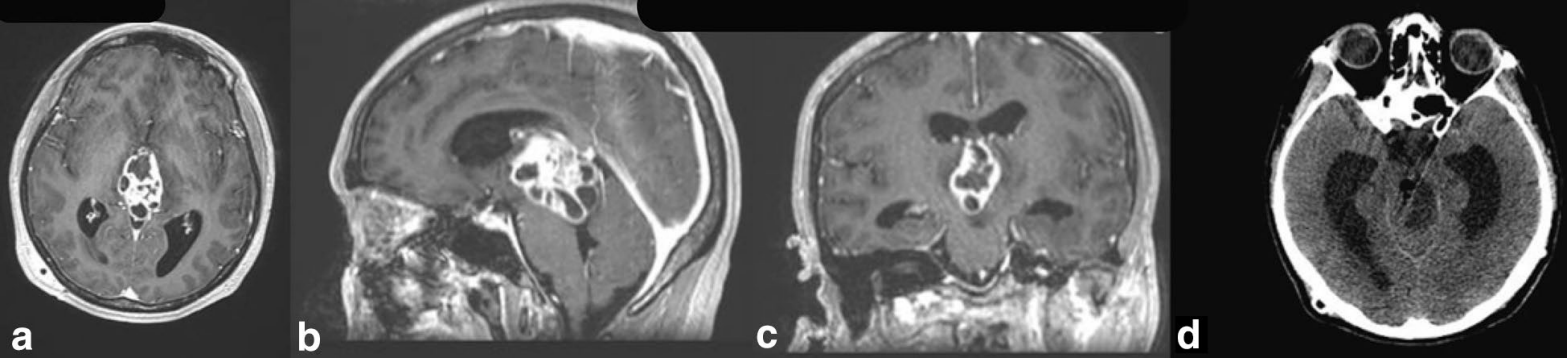
A multi-cystic heterogeneously enhanced mass is observed in the third ventricle on T1-weighted brain MR (**a**–**c**). Temporal horns are dilated despite previous V-P shunt surgery (**d**)

**Fig. 2:**
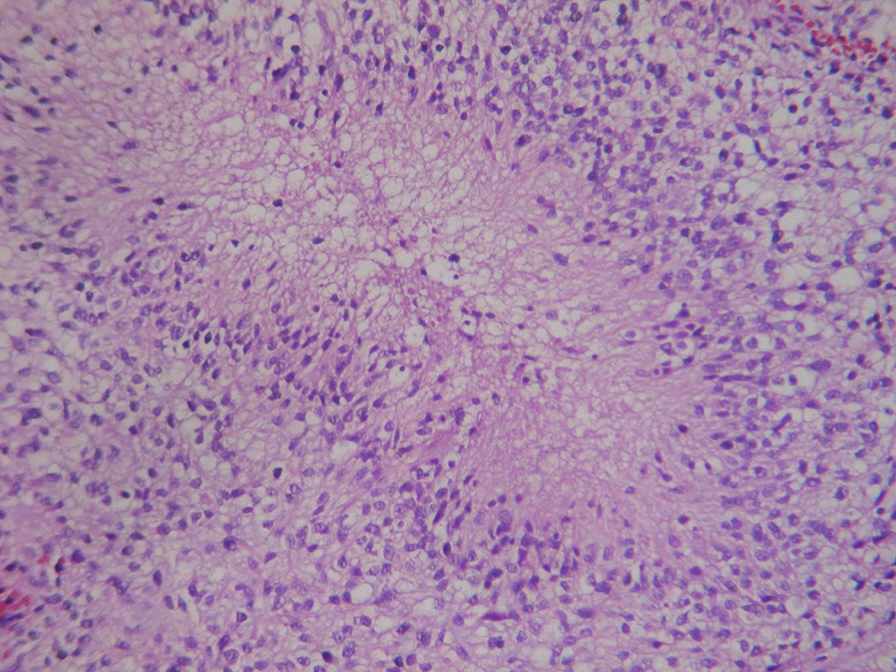
Histopathological appearance of the tumor (H&E ×200)

**Fig. 3:**
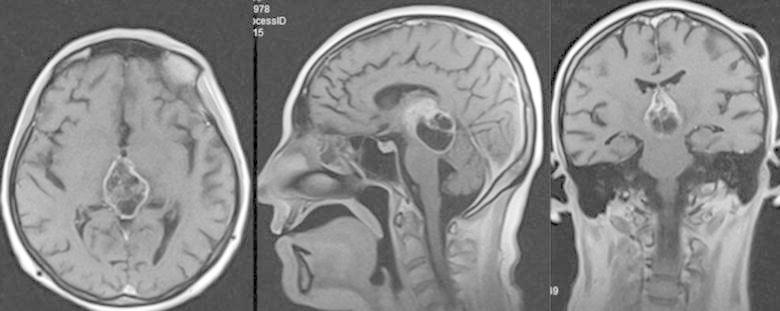
Post-operative 6th month brain MRI depicts resolution of hydrocephalus and more necrotic consistency of the lesion

In her examination, there was cognitive decline and gait instability. A heterogeneously enhanced multi-cystic isolated third ventricular neoplasm was observed on the brain MRI (Fig. 1a–c). We also noticed that hydrocephalus had not completely resolved with previously inserted V-P shunt (Fig. 1d). A stereotactic biopsy from the mass lesion and insertion of a second V-P shunt from the opposite site were managed in the same surgical session. The surgery was successful without any complication. Frozen-section result was compatible with high-grade glioma. Post-operative course of the patient was uneventful. Histopathological diagnosis was glioblastoma (grade 4 glioma) (Fig. 2). There were moderate amount of tumor cells composed of ovoid and round astrocyte-like cells. Mitosis activity was 6/10 high power fields. Vascular endothelial proliferation was present with necrosis in palisading nature. Immunohistochemical staining depicted GFAP+, Olig2+, EMA−, synaptophysin−, Ki67 index of 45 %. Both radiotherapy and chemotherapy were conveyed after the final diagnosis. The patient’s general status was well 6 month after the biopsy, the hydrocephalus has resolved and the lesion has become more necrotic in consistency (Fig. 3).

## Discussion

Gliomas are the most common primary tumors of CNS in adult patients. Glioblastoma is the most malignant and most common type (50–60 %) of all gliomas (Prieto et al. 2006). Glioblastoma incidence has been reported between 0.59 and 3.69 in 100,000 persons (Ostrom et al. 2015). Usual tumor location for gliomas is cerebral hemispheres. Presentation of glioblastoma solely in the third ventricle is very rare (Prieto et al. 2006; Lee and Manzano 1997; Hariri et al. 2014; Vougiouklakis et al. 2006; Lejeune et al. 2000; Villani and Tomei 2000; Yasargil 1996; AlbertLasierra 1999).

Magnetic resonance imaging is useful for both diagnostic and therapeutic aspects. Before ascribing glioblastoma as pure 3rd ventricle neoplasm, its possible interdigitating nature with surrounding brain parenchyma (thalamus, caudate nucleus) should be carefully inspected on MRI (Prieto et al. 2006). Third ventricular glioblastoma may have arised de novo or may have upgraded from a former low-grade glioma in the same location (Prieto et al. 2006; Lee and Manzano 1997; Vougiouklakis et al. 2006; Pasquier et al. 2002). Origin for the tumors without any proof of metastasis from other sites (as in this case) can be subependymal glial tissue surrounding the third ventricle (Prieto et al. 2006; Lee and Manzano 1997).

In differential diagnosis list of the tumors presenting in the third ventricle, there are plenty of tumors such as colloid cyst, meningioma, germinoma, craniopharyngioma, lymphoma, choroid plexus papilloma, subependymal giant cell astrocytoma, chiasmatic and hypothalamic benign astrocytoma (Lee and Manzano 1997). Ring enhancement is a peculiar sign for glioblastoma, yet not pathognomonic (Deramond et al. 2000). Tumor histology is crucial to yield the final diagnosis.

Total resection is possible yet hard due to very deep location of the tumor at the center of the brain, within surrounding delicate tissues and fragile vessels. Malignant nature and intractable course of the tumor should be considered while planning treatment. Lee and Manzano (1997) mentioned short survival time of their patient after gross tumor resection. If surgical excision is still considered, endoscopic approach is superior to other approaches in respect of direct tumor visualization (Gaab and Schroeder 2000). Endoscopic approach to the third ventricle is a minimally invasive procedure compared to conventional craniotomy. By this approach not only access to the tumor itself, but also relief of the hydrocephalus caused by the tumor could be managed simultaneously using ventriculostomy, septostomy or aqueductal stenting (Schroeder 2013). Operative time and morbidity rates attributed to endoscopic approach to third ventricle are lower than microsurgical approach. However, complete resection rate is higher in microsurgical approach by using two-handed techniques during the procedure (Ibanez-Botella et al. 2014; Sheikh et al. 2014). Reported complications of endoscopic approach to the 3rd ventricle are hemorrhage (the most common), CSF leaks, meningitis, subdural hematoma, memory problems (Schroeder 2013). Tumor size is a significant indicator for endoscopic approach preference. Due to eloquent nature of the 3rd ventricle, total resection via endoscopic approach could not be managed directly; instead peace-meal resection is preferred. Peace-meal resection could take more time in large-sized tumors, which might make endoscopic approach less favorable in this respect. Even though there is no one widely accepted threshold for tumor size, Schroeder (2013) proposed it as 2 cm. These results have been retrieved from large series of other kind of pathologies residing purely in the 3rd ventricle. Even though it is impossible to make similar conclusions for 3rd ventricle glioblastomas since they are rare in this location (Table 1), it is logical to expect similar issues to be faced against. In the present case, the patient had already been operated with V-P shunt before admission to our clinic. However, there was recurrent hydrocephalus and the tumor was huge and infiltrating whole of the 3rd ventricle (Fig. 1a–c). Ventricular tip of the prior V-P shunt had been put into the right occipital horn. With the second V-P shunt, the third ventricle was by-passed concomitant with stereotactic biopsy of the tumor. Definitive diagnosis was glioblastoma with Ki67 index as high as 45 %. Radiotherapy and chemotherapy were started immediately. The patient’s general status was well 6 months after the biopsy, the hydrocephalus has resolved and the lesion has become more necrotic in consistency.

## Conclusion

Presentation of glioblastoma as an isolated mass in the third ventricle is very rare. Although MR is very useful in detection of the lesion, definitive diagnosis depends on histopathology due to many other different neoplasms that may present in the third ventricle. Management of obstructive hydrocephalus and giving radiotherapy with chemotherapy after histopathological diagnosis is safe and effective therapeutic approach in isolated third ventricular glioblastoma.
